# Anti-oxidative effect of zinc in human umbilical cord mesenchymal stem cells

**DOI:** 10.52601/bpr.2021.200046

**Published:** 2021-04-30

**Authors:** Xiaodan Lu, Yifan Lin, Xiuying Lin, Qiang Zhang, Zihang Wang, Xuguang Mi, Ruobing Wang, Xiaofang Zhang, Xu Luan, Yan Liu, Bing Li, Yan Tan, Yanqiu Fang

**Affiliations:** 1 Diagnostic Medical Center, Jilin Province People's Hospital, Changchun 130021, China; 2 School of Medicine, Changchun University of Chinese Medicine, Changchun 130021, China; 3 Department of Clinical Laboratory, the Second Hospital of Jilin University, Changchun 130041, China; 4 General Surgery, the First Hospital of Jilin University, Changchun 130021, China

**Keywords:** Zinc, Oxidative stress, Nuclear factor erythroid-2-related factor 2, Sirtuin 3, Human umbilical cord mesenchymal stem cells

## Abstract

Human umbilical cord mesenchymal stem cells (HUC-MSCs) are pluripotent and functional in many biological processes, by which releasing secretary factors to promote the self-repairing of damaged tissue or developing into functional cell at local organ. However, there is a high risk that oxidative stress would reduce the pluripotency and factor-secretion during the preparation and transplantation. Therefore, reducing oxidative stress is expected to improve the efficacy of HUC-MSCs therapy. Zinc (Zn) is an essential trace element which involves in the resistance of oxidative stress. To investigate Zn-regulated signaling pathways, we have profiled the gene expression at transcriptome level in primary HUC-MSCs treated with zinc sulfate, followed with GO and KEGG gene enrichment analysis. Zn treatment improved signal pathways for mineral absorption, cell growth, and cell death. Zn deficiency was mimicked by TPEN administration, which suppressed cell proliferation and reduced the expression of HUC-MSCs surface stem cell markers CD73, CD90 and CD105 by flow cytometry. Nuclear factor erythrocyte 2 related factor 2 (Nrf2) plays an important role in antioxidant biological processes. *In vitro* treatment of Zn significantly increased Nrf2 and Sirt3 expression at gene level and protein level respectively. Zn supplementation inhibited TPEN-induced failure of cell survival and reversed the reduction of Nrf2 and Sirt3 expression, which further reduced the production of ROS. Zn successfully presented its anti-oxidation effect by activating Nrf2/Sirt3 signaling pathway in HUC-MSCs. Zn supplementation may improve the efficacy of HUC-MSCs therapy with reduced oxidative stress.

## INTRODUCTION

Human umbilical cord mesenchymal stem cells (HUC-MSCs) are pluripotent with self-renewal potential and capability of expansion *in vitro* (Vohra *et al*. 2020). They are able to differentiate into various functional cell types under certain conditions. In addition, human umbilical cord tissue seems to be considered the best resource of adult stem cells (Ding *et al*. 2015). There are a large number of clinical trials to explore HUC-MSCs for treatment of various diseases, such as autoimmune diseases, diabetes, bone defects, myocardial infarction, and even in COVID-19 infections (Camilletti *et al*. 2020; Hubber *et al*. 2021; Park *et al*. 2021; Yang *et al*. 2021; Ziaei *et al*. 2017). HUC-MSCs have become increasingly popular, due to their efficiency in homing to tissue injury sites, differentiation potential, secretary factors, and immuno-regulatory effects, which are expected to be one of the promising therapeutic treatments (Li *et al*. 2020; Wang *et al*. 2020b; Yao *et al*. 2019). It is speculated that at the ischemic site of injury, ROS and non-specific inflammation will repress HUC-MSCs transplantation efficiency. 17β-Estradiol is able to protect mesenchymal stem cells against high glucose-induced mitochondrial oxidants production (Oh *et al*. 2019). Therefore, there is an urgent need to identify a method to reduce the oxidative stress in the MSCs-injury contact microenvironment, which will further effectively promote the transplantation and repair.

Zinc (Zn) is an important trace element, which involved in a variety of biological functions such as DNA synthesis, cell division, gene expression and the regulation of innate immune response (Gammoh and Rink 2017; Ma *et al*. 2020). Zinc deficiency is closely associated with respiratory infections, diarrhea, and dermatitis in pediatric practice (Gammoh and Rink 2017; Sanna *et al*. 2018). Zinc deficiency increases inflammation by elevating inflammatory response and damages to the host tissue. Many studies have demonstrated a link between zinc deficiency and the development of oxidative stress (Millward 2017). In our previous study, we have discovered that lowered Zn-level is related with female infertility. Zn is able to active anti-oxidative effect through the Nuclear factor erythrocyte 2 related factor 2 (Nrf2)/PGC-1α pathway in human endometrial cells (Lu *et al*. 2020). Zn plays anti-apoptotic role in type 2 diabetic nephropathy through Wnt/β-catenin signaling pathway (Hadj Abdallah *et al*. 2018; Wang *et al*. 2020a).

Nrf2 is a key nuclear transcription factor, which playing regulatory roles in anti-oxidative stress and cellular redox homeostasis (Marchev *et al*. 2017). Sirt3 is a member of Sirtuin family of class III histone deacetylases (Hirschey *et al*. 2010). Diseases associated with SIRT3 include aging and non-alcoholic fatty liver disease (Katwal *et al*. 2018). It is a key regulator of the mitochondrial respiratory chain and functional in inhibiting oxidative stress. Transcription factor Nrf2 could bind to the promoter region and participate in regulating Sirt3 expression (Wang *et al*. 2017). However, the environmental oxidative stress-induced defects are still unclear in the application of HUC-MSCs. In this study, we aimed to investigate zinc depletion and zinc supplementation on redox-relevant systems within HUC-MSCs.

## RESULTS

### Antioxidant effect of zinc on HUC-MSCs

**Figure 1:**
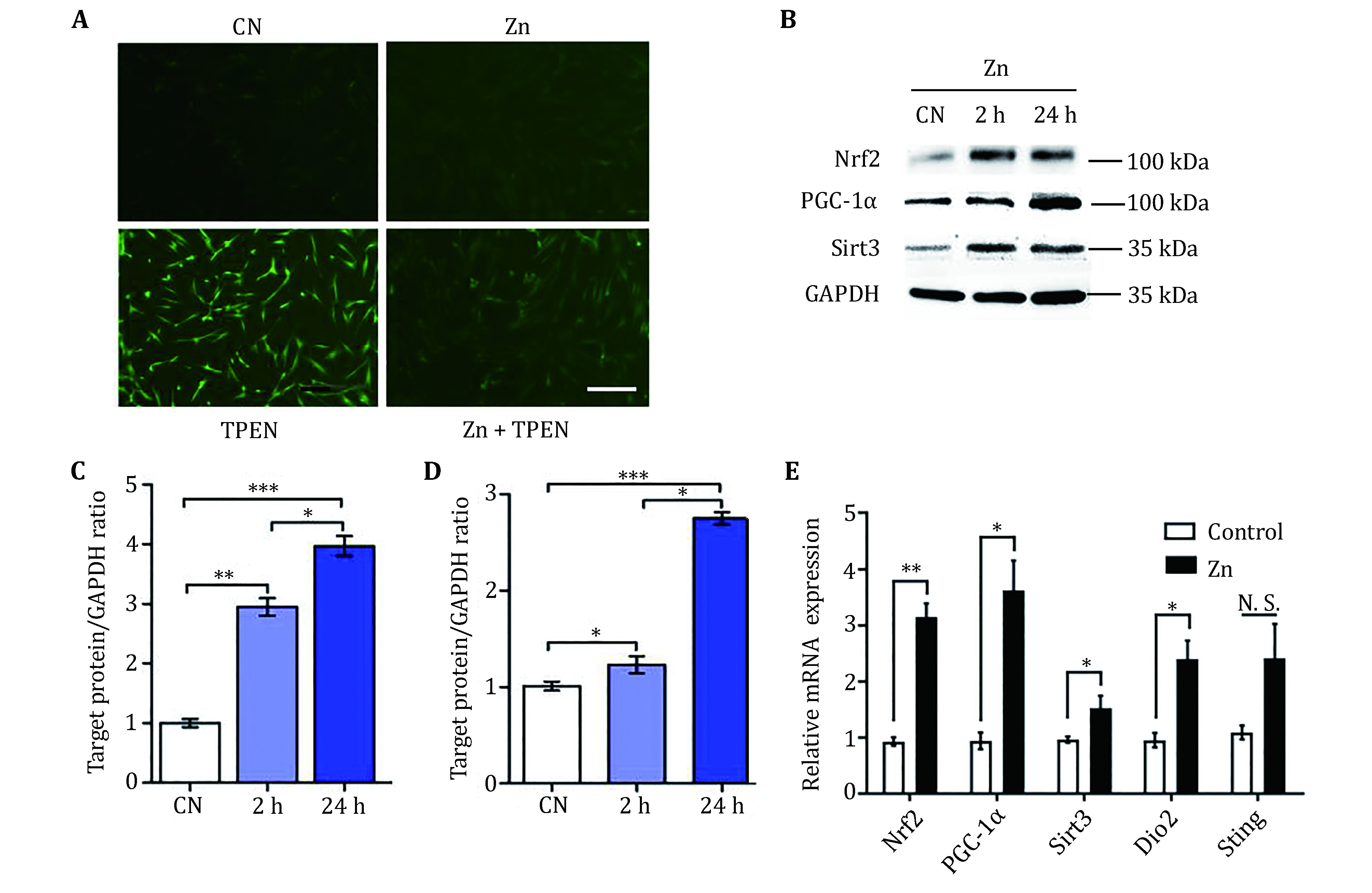
Antioxidant effect of Zn on HUC-MSCs through Nrf2/Sirt3 signaling pathway. **A** The effect of Zn on the level of ROS production. DCFH-DA staining was used to measure intracellular ROS production. The scale bar indicates 100 μm. **B** Over time, under zinc treatment, the expression of Nrf2, Sirt3, and PGC-1α protein was measured by an illustrative Western blot. Four independent experiments were carried out and typical results were given. **C, D** Normalized the quantification of Nrf2 (**C**) and PGC-1α (**D**) to GAPDH and measured by image J. The results are expressed as mean ± S.D., *p < 0.05, **p < 0.01, ***p < 0.001. **E** Real-time quantitative PCR results of Nrf2, Sirt3 and PGC-1α gene expression under Zn treatment (*n* = 5). *p < 0.05, **p < 0.01

Zn is an essential trace element which is necessary for human health. Here, we employed the zinc chelator N,N,N,N-Tetrakis(2-pyridylmethyl)-ethylenediamine (TPEN) to mimic zinc deficiency in HUC-MSCs. TPEN is a specific cell-permeable heavy metal chelator that can induce ROS in HUC-MSCs, detected by DCFH-DA staining (Fig. 1A). Zinc treatment attenuates TPEN-induced ROS production. Zinc plays an antioxidative role in HUC-MSCs. In addition, we measured the expression of antioxidant signal-related genes *Nrf2* and *PGC-1α* in umbilical cord mesenchymal stem cells. The protein expression of Nrf2 and PGC-1α increased within 2 h (**p < 0.01) and significantly increased within the next 24 h (***p < 0.001), which was quantified by Image J (Fig. 1B, C and D). Consistent with this finding, PCR results showed that after treatment of umbilical cord mesenchymal stem cells with Zn for 24 h, the expressions of Nrf2, PGC-1α, and Sirt3 increased significantly (*p < 0.05) (Fig. 1E). After 24 h of TPEN treatment, Nrf2, PGC-1α, and Sirt3 were significantly reduced (*p < 0.05).

**Figure 2:**
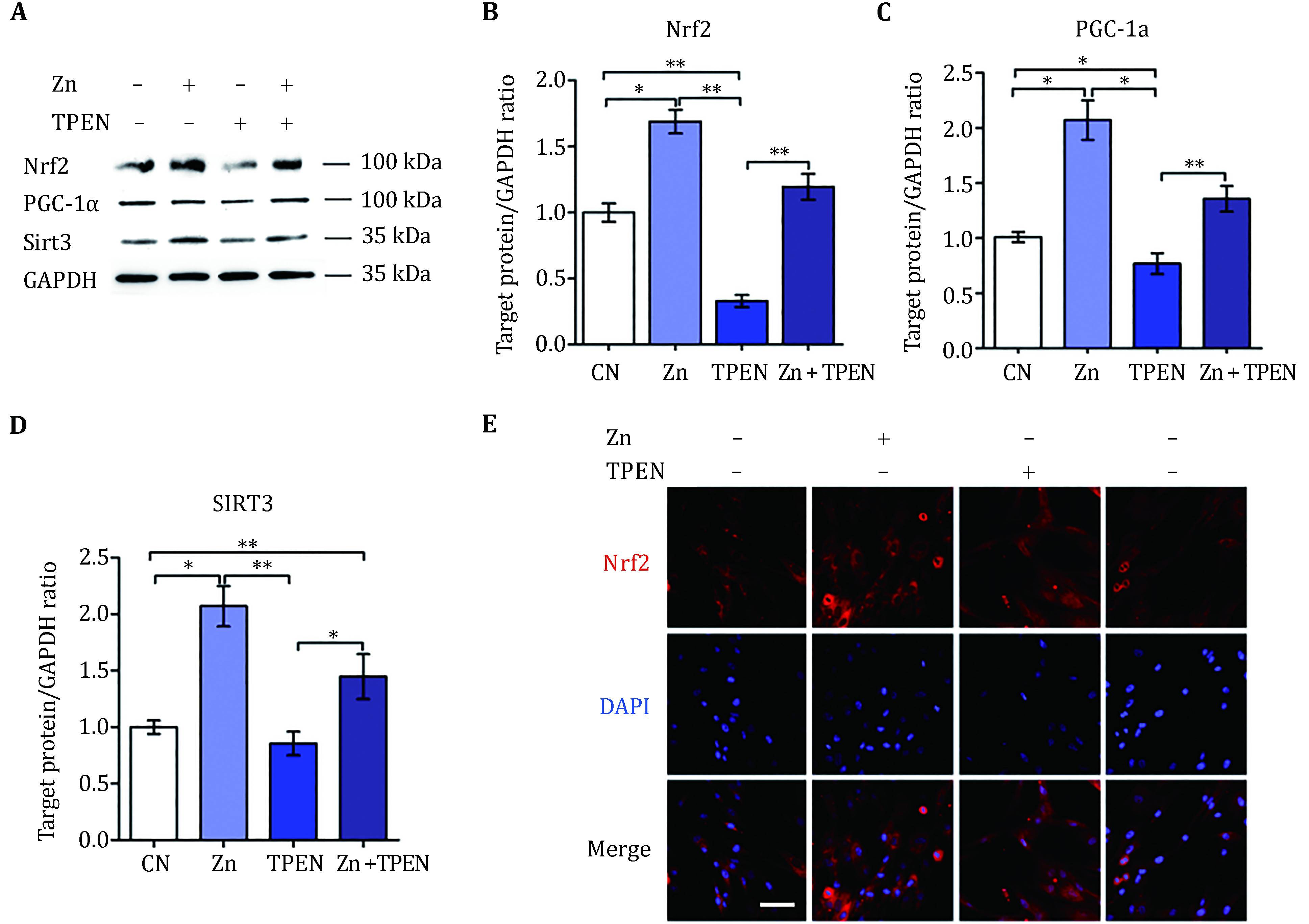
Zinc regulated Nrf2 expression in HUC-MSCs. **A** Illustrative western blotting measured Nrf2, PGC-1α and SIRT3 protein expression. Four independent experiments were implemented and the typical results are presented. **B, C, D** Quantification of Nrf2 (**B**), PGC-1α (**C**) and SIRT3 (**D**) were normalized to GAPDH and measured by Image J. Results are presented as mean ± S.D. *p < 0.05, **p < 0.01. **E** Immunofluorescent staining of Nrf2 (red) in HUC-MSCs. DAPI was used for nucleus staining (blue). The scale bar indicates 50 μm

Zinc pretreatment was able to reverse the TPEN-induced reduction of Nrf2 and SIRT3 expression (Fig. 2A). In the quantification by Image J, the expressions of Nrf2 and SIRT3 were significantly increased compared to control group and TPEN-treated group (Fig. 2B, C and D). In addition, the effect of Zn on the expression and subcellular localization of Nrf2 in the cell compartment was examined by Nrf2 immunofluorescence staining. Nrf2 expression was suppressed in TPEN-treated cells, but significantly increased in Zn-treated cells, and the TPEN effect was completely eliminated by Zn treatment (Fig. 2E). It is worth noting that after adding Zn pretreatment, the fluorescence intensity in the cytoplasm and nucleus increased significantly, which indicated that the addition of exogenous Zn is able to overcome the reduction of TPEN-induced zinc deficiency.

### Zn is essential for cell survival of HUC-MSCs

**Figure 3:**
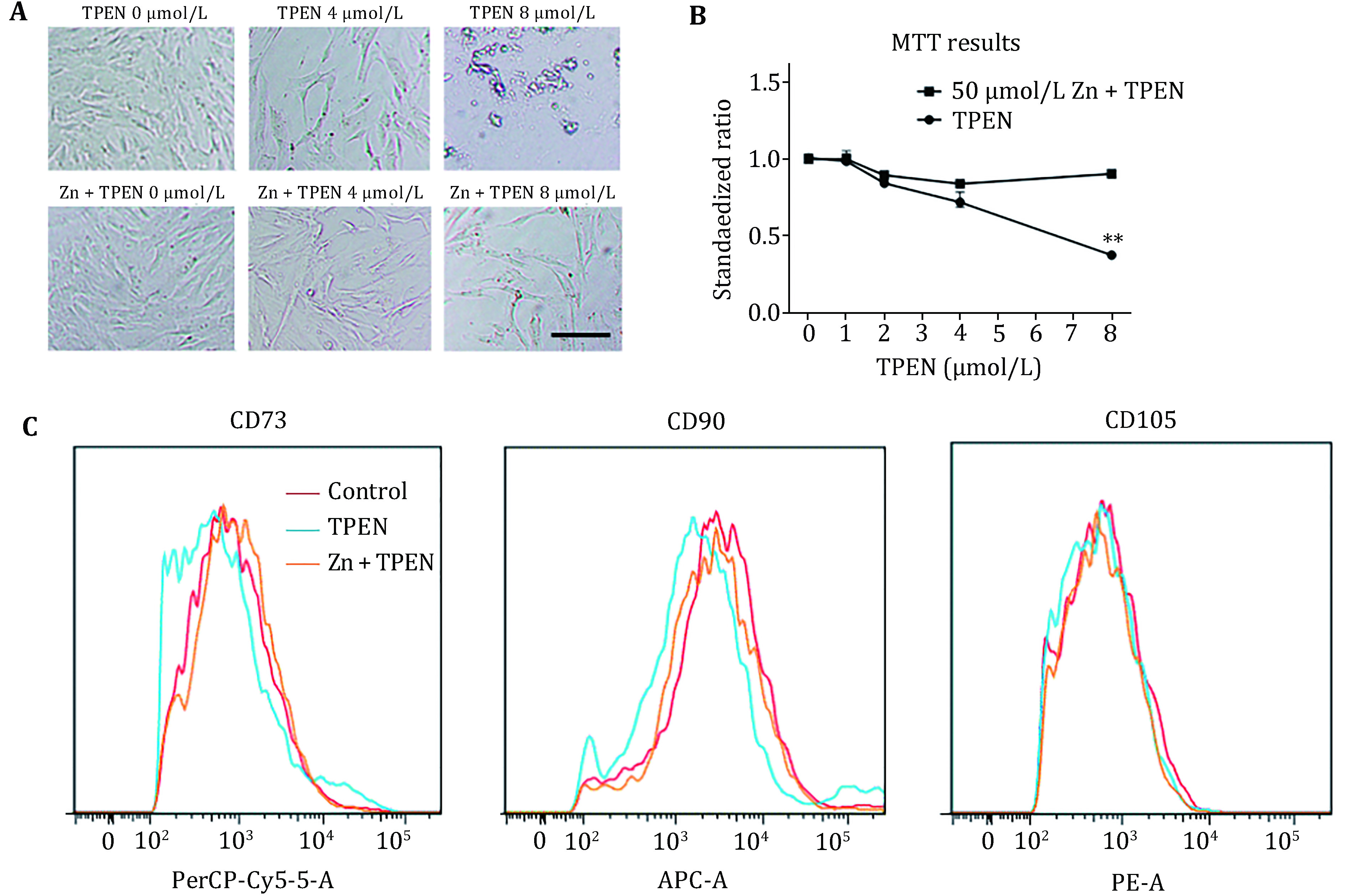
TPEN-induced Zn deficiency in HUC-MSCs. The cells were treated by TPEN or Zn with TPEN for 24 h. **A** Cell culture image of HUC-MSCs. The scale bar represents 50 μm. **B** Cell survival rate were measured by MTT assay. **p < 0.01. **C** Stem cell marker CD73, CD90, CD105 positive cells were calculated by flow cytometry. Data are representative of four independent experiments

Cells were treated with TPEN or 50 μmol/L Zn plus TPEN for 24 h, dose-dependently. The cells were died under 8 μmol/L TPEN treatment, but successfully survived by exogenous Zn supplement in both of the cell culture system and MTT assay significantly (Fig. 3A, B). The HUC-MSCs are functional in their pluripotency and express stem cell-specific markers. Here we measured CD73, CD90, and CD105 positive HUC-MSCs by flow cytometry (Fig. 3C, D, E). TPEN attenuated CD73 and CD90 expression in HUC-MSCs when CD105 positive cells were not affected by TPEN. In a contrast, exogenous zinc supplementation effectively recovered the expression of CD73 and CD90 in HUC-MSCs. Taken together, our results indicate that Zn is essential for cell survival and the pluripotency of HUC-MSCs.

### Transcriptome profiling of zinc-induced effect on HUC-MSCs

**Figure 4:**
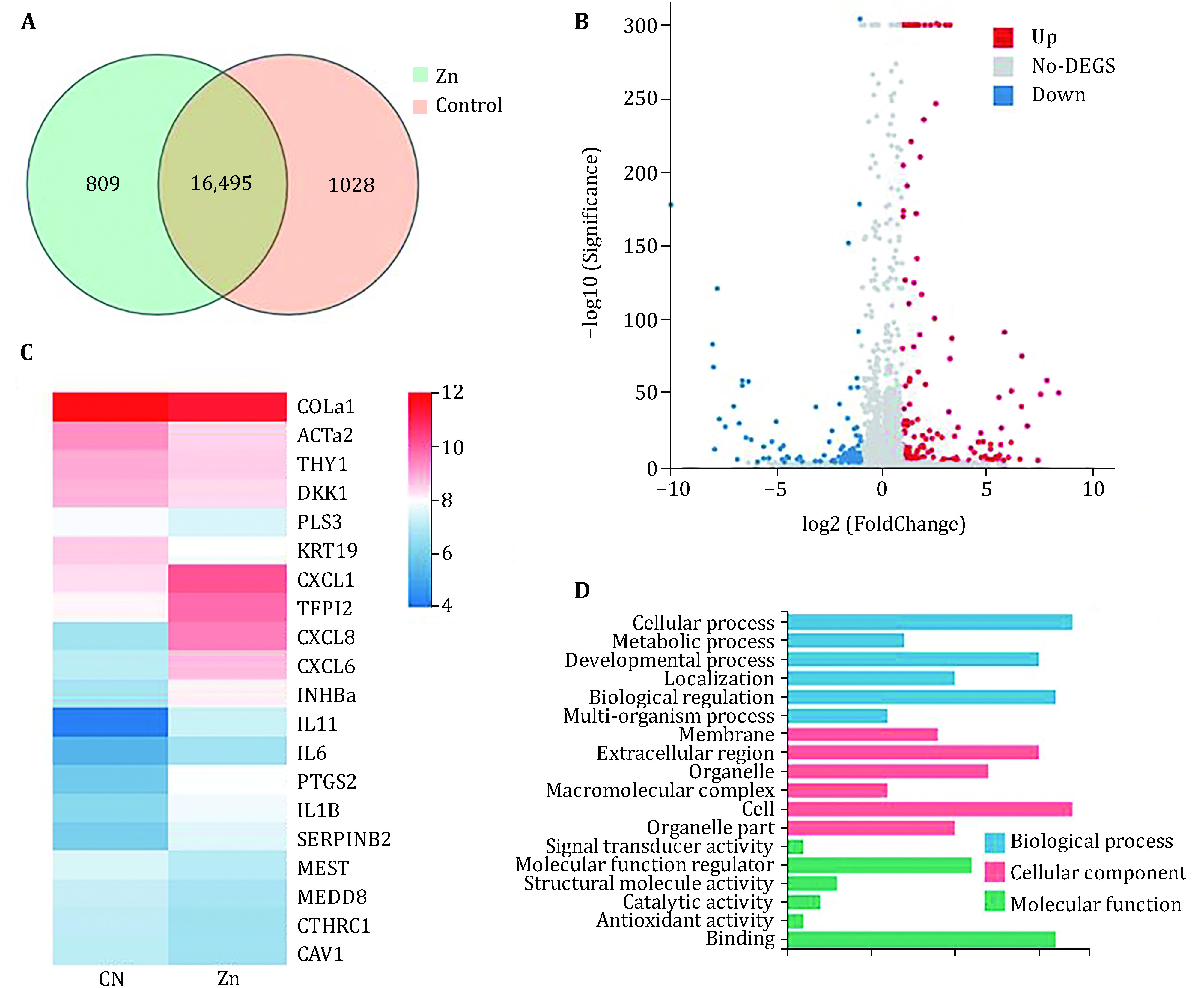
Transcriptional profiling of Zn regulated genes in HUC-MSCs. The cells are treated with Zn (50 μmol/L) for 24 h (*n* = 6). **A** Venn diagrams present specifically expressed gene number. **B** Volcano plot indicates up-regulated genes in red and down-regulated genes in blue. **C** Heatmap presents genes with most significant changes after Zn treatment. **D** GO analysis of gene enrichment in HUC-MSCs

To investigate the key signaling pathways by zinc regulation, transcriptomics was used in our experiment. A total of 18,332 genes were detected in HUC-MSCs, 16,495 genes were commonly expressed, while 809 genes were specifically responded to Zn treatment (Fig. 4A). In addition, 268 genes were specified with significant differences after Zn treatment, with FPKM over 100 and the *p* value less than 0.05. It contains 157 up-regulations and 111 down-regulations (Fig. 4B). The top 20 of genes with the most significant changes were shown in the heatmap (Fig. 4C). With Zn treatment, IL11, CXCL8, PTGS2, CXCL1, TFPI2, CXCL6, IL1B, IL6, INHBα, PLS3 expression were elevated, and CTHRC1, THY1, DKK1, MEST, COL1α1, KRT19, ACTα2, MEDD8, CAV1, SERPINB2 expression were decreased significantly (*p* < 0.05). Genes with FPKM values ​​greater than 100 copies were used for gene oncology (GO) pathway enrichment analysis (Fig. 4D). Preliminary identified biological processes are cellular processes, metabolic progress, developmental progress, response to stimuli and biological regulation. Molecular functions include binding, structural molecular activity, transporter activity, molecular function regulators and catalytic activity.

**Figure 5:**
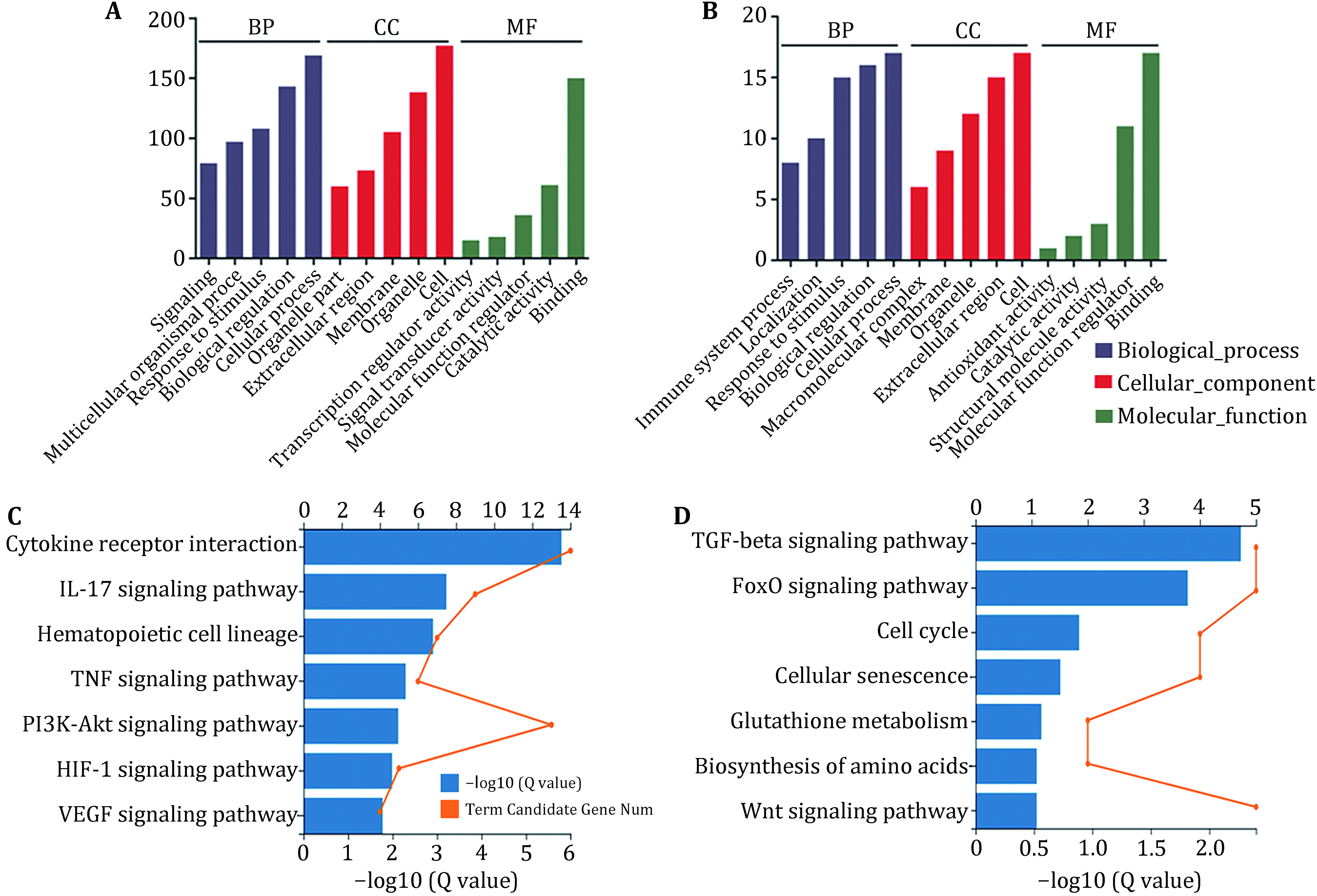
GO and KEGG analysis of genes up- and down-regulated by Zn. **A, B** GO classification of genes up-regulated (**A**) and down-regulated (**B**) by Zn. **C, D** KEGG analysis of genes up-regulated (**C**) and down-regulated (**D**) after Zn treatment. Genes expression higher than 200 copies and the corrected *p* value (Q value) < 0.05 were screened, which was based on the Kyoto Encyclopedia of Genes and Genomes (KEGG) database

GO analysis showed that among the up-regulated genes of human umbilical cord mesenchymal stem cells, the most abundant up-regulated genes are involved in signal transduction, multi-cellular biological processes, responses to stimuli, biological regulation, cellular processes, organelle parts, extracellular regions, membranes, organelle, cell, transcriptional regulator activity, signal transducer activity, molecular function regulator, catalytic activity and binding (Fig. 5A). KEGG pathway enrichment showed that the most abundant up-regulated genes are involved in the cytokine receptor interaction, IL-17 signaling pathway, hematopoietic cell lineage, TNF signaling pathway, PI3K-Akt signaling pathway, HIF-1 signaling pathway and VEGF signaling pathway (Fig. 5C). Among the down-regulated genes, GO analysis showed that the most abundant down-regulated genes are involved in immune system processes, localization, response to stimuli, biological regulation, cellular processes, macromolecular complexes, membranes, organelles, extracellular regions, cells, antioxidant activity, catalytic activity, structural molecular activity, molecular function modifiers and binding (Fig. 5B). KEGG pathway enrichment showed that the most abundant down-regulated genes in human umbilical cord mesenchymal stem cells are involved in the TGF-beta signaling pathway, FoxO signaling pathway, cell cycle, cellular senescence, glutathione metabolism, biosynthesis of amino acids and Wnt signaling pathway (Fig. 5D).

## DISCUSSION

In this study, we revealed that Zn could increase antioxidative stress properties by directly up-regulating the Nrf2/Sirt3 signaling pathway in HUC-MSCs for the first time. The depletion of Zn leads to repression of Nrf2 and Sirt3 in both gene and protein levels. Therefore, Zn is essential for the survival and differentiation potentials of HUC-MSCs. MSCs are adult stem cells found in bone marrow, umbilical cord blood, adipose tissue, and many other tissues, which are easy to be collected and possess immunogenicity, self-renewal and multidirectional differentiation ability (Ding *et al*. 2015; Roura and Vives 2019). Nowadays, MSCs-based methods, especially HUC-MSCs have become a promising treatment in various diseases (Oggu *et al*. 2017; Su *et al*. 2019). Maintaining redox homeostasis plays a vital role in regulating the self-renewal and differentiation of HUC-MSCs (Song *et al*. 2018). The accumulation of oxidative stress and the imbalance of key differentiation regulators determine the differentiation potential of HUC-MSCs. It is important to develop strategies to reduce oxidative stress and maintain the viability and differentiation potential of MSCs. Therefore, Zn supplementation in HUC-MSCs transplantation could significantly reduce the level of oxidative stress and achieve effective therapeutic effects.

Zn is a nutrition-related micronutrient that is essential for the structure and function of many macromolecules, including enzymes and cellular processes that regulate cellular signaling pathways (Benameur *et al*. 2015). It is reported that Zn deficiency increases the production of reactive oxygen species (ROS) and inflammatory cytokines (such as TNF-α, IL-1β, IL-8, VCAM and MCP-1). Zn can be an effective antioxidant and anti-inflammatory drug and proper zinc supplementation can reduce the occurrence of oxidative stress and inflammatory reactions (Ohashi and Fukada 2019). Our results on transcriptome profiling have revealed up-regulated and down-regulated genes after Zn treatment. The C-X-C motif chemokine ligands CXCL 1, 6, 8 are over-expressed in Zn-group, which indicates the HUC-MSCs responses to zinc treatment and triggers other downstream signaling pathways. The CXC chemokines are closely related to angiogenesis in health and disease (Rosenkilde and Schwartz 2004). Many cellular signaling pathways are up-regulated after Zn treatment, which may be responsive to the CXC chemokine signals. TGF-β signaling pathway is down-regulated in KEGG pathway analysis. Wnt signaling is slightly decreased, which is not consistent with our previous study on zinc-induced Wnt/β-catenin up-regulation in type 2 diabetic nephropathy (Wang *et al*. 2020a). It suggests that in difference condition or cell types, zinc may act in different ways.

Oxidative stress and inflammation are inseparable and are the mediators of the development of disease and its complications. Studies have shown that oxidative stress associated with cell therapy could increase production of ROS and reduce antioxidant capacity (Afshar Ebrahimi *et al*. 2018). The latter is caused by impaired activation of Nrf2, a transcription factor that regulates genes encoding antioxidants. Nrf2 transcription pathway is the main cellular defense system, mainly pro-oxidative and pro-inflammatory stress (Li *et al*. 2018). In general, Nrf2 regulation provides the interface between redox and intermediate metabolism (Kumar *et al*. 2020). The interaction between Sirt3 and oxidative stress has been documented. It has been described that mitochondrial Sirt3 controls the flow of mitochondrial oxidation pathways and thus controls the rate of active oxygen production (Farruggia *et al*. 2018). Sirt3-mediated deacetylation activates the enzyme responsible for quenching ROS, providing deep protection against oxidative stress-related diseases (Liu *et al*. 2017). Our data indicate that high doses of zinc can induce changes in the Nrf2/Sirt3 signaling pathway in umbilical cord mesenchymal stem cells, thereby increasing their ability to resist oxidative stress. In addition, our results of transcriptome profiling data have shown that Zn significantly regulated the developmental progress, response to stimuli and biological regulation, antioxidant activity and longevity regulatory pathway in the transcriptional level, which are closely related to the MSCs development and implantation.

Therefore, HUC-MSCs therapy with zinc sulfate as adjuvant could develop into a new approach in the field of stem cell therapy. The development of such therapies requires continued efforts by the research community to better understand the effects of oxidative stress on mesenchymal stem cells.

## MATERIALS AND METHOD

### Subjects

This study was approved by ethics committee of Jilin Province People's Hospital. Pregnant women who admitted to the Obstetrics Department of Jilin Province People's Hospital signed written informed consent to participate in this study and assisted in the improvement of each pregnancy check during the period from January 2017 to December 2018. The newborn's umbilical cord is taken about 10 cm in the full-term production of the participants, and transported from the delivery room to the laboratory under sterile conditions for subsequent experimental studies. All participants were normal pregnancy, and have no history of infectious disease, no history of smoking and alcohol abuse. All umbilical-derived newborns are healthy and alive.

### Cell culture and treatment

Neonatal umbilical cords were collected and placed in sterile phosphate buffered saline (PBS) (Invitrogen Carlsbad, CA, USA) and transferred to the laboratory with ice transport. Umbilical cord tissue was taken under sterile conditions, washed with PBS; amniotic membrane was removed, umbilical cord was removed longitudinally to remove veins and arterial vessels; Wharton matrigel was separated, and the colloid was cut into 1 mm^3^ tissue blocks; tissue blocks were inoculated into culture dishes at intervals of 3–5 mm. Adherently, carefully add 6 mL of MEM medium containing 10% heat-inactivated fetal bovine serum (Hyclone, Logan, USA) and antibiotic with penicillin and streptomycin (Invitrogen, Carlsbad, CA) at 37 °C and 5% CO_2_ concentration. Human primary umbilical cord mesenchymal stem cells were seeded at a density of 0.1×10^4^ cells or 1×10^5^ cells per well, respectively. The 50 μmol/L Zn sulfate, 4 μmol/L Zn-specific and membrane-permeable zinc chelator TPEN or 50 μmol/L Zn supplemented with 4 μmol/L TPEN treatment was administrated *in vitro*.

### Flow cytometry

Umbilical cord mesenchymal cells were stimulated with zinc or additional TPEN for 24 h. Cells were digested with 0.25% trypsin, washed three times with saline, and perCP-Cy5.5-anti-CD73 (BD Biosciences, San Diego, California, 561260), APC-anti-CD90 (BD Biosciences, San Diego, California, 740585), PE-Anti-CD105 (BD Biosciences, San Diego, California, 562380) protected from light for 20 min at room temperature (25 °C) after antibody. For each sample, 10,000 cells were collected and measured by FACS Calibur (BD Biosciences). FlowJo 10.4 software was used for data analysis.

### ROS assay

ROS levels in umbilical cord mesenchymal stem cells were measured using a 2,7-dichlorodihydrofluorescein-diacetate kit (DCFH-DA) (Beyotime, S0033, China). The DCFH-DA probe was diluted 1:1000 to a final concentration of 10 μmol/L in serum-free DMEM medium. Umbilical cord mesenchymal stem cells were seeded into 6-well plates. After treatment, cells were incubated with DCFH-DA at 37 °C for 20 min. After washing three times with serum-free DMEM, the cells were imaged by a fluorescence microscope (Leica DMI3000B, Germany). Experiments were performed in triplicate.

### RNA-seq analysis

RNA extraction, library preparation, RNA-seq and bioinformatics analysis of human umbilical cord mesenchymal stem cells were performed at BGI (Beijing Genomics Institute, Shenzhen). The sequencing reads were compared to the human genome (GCF_000001405.37_GRCh38.p11) and used to calculate the gene count representing the total number of sequencing reads compared to the genes. The DESeq2 algorithm was used to identify differentially expressed genes between control samples and zinc-treated samples. The BGI Dr. Tom operating system was used for KEGG pathway enrichment and gene ontology analysis.

### MTT assay

The laboratory measured cell viability by 3-(4,5-dimethylthiazol-2-yl)-2,5-diphenyltetrazole bromide (MTT) (Sigma-Aldrich, UK) assay. Umbilical cord mesenchymal stem cells were seeded in 96-well plates overnight and then treated with Zn or TPEN for 24 h. MTT were treated at 5 mg/mL final concentration and incubate at 37 °C for 4 h. After removing the supernatant containing MTT, dissolved the remaining crystal precipitate in 150 μL dimethyl sulfoxide and used a microplate reader (Multiskan Ascent Thermo Scientific, UK) measured the absorbance at 570 nm.

### Western blot

Cells were lysed in RIPA buffer (50 mmol/L Tris-HCl, 0.1% SDS, 150 mmol/L NaCl, 1% Triton X-100, 1% sodium deoxycholate and protease inhibitor, P0013, Beyotime) on ice. After centrifugation at 13,300 r/min for 15 min at 4 °C, the cell lysate was collected. Protein concentration was quantified by BCA protein assay kit (Beyotime, P0011, China). 20 μg of protein was loaded on an SDS-PAGE gel for separation, and then transferred to a PVDF membrane. After blocking for 1 h in TBST containing 5% non-fat milk at room temperature, the membrane was incubated with primary antibody at 4 °C overnight. Followed with secondary antibody for 2 h at room temperature. The membrane was imaged with a chemiluminescence detection kit (TransGen Biotech, Beijing, China) using an enhanced chemiluminescence system (Tanon Biotech, Shanghai, China). The intensity of the band is quantified by the Image J software.

Mouse primary antibody against human GAPDH (Santa Cruz, sc-166574; 37kD; 1:2000), rabbit anti-human Nrf2 (Abcam, 100kD; 1:1000), rabbit anti-human Sirt3 (Abcam, 35kD; 1:1000) and rabbit anti-human PGC-1α (Abcam, 100kD; 1:1000) were used. The secondary antibody is combined with HRP IgG antibody (Cell signal technology, 7074S/7076S; 1:2000).

### RT-qPCR analysis

Total RNA was isolated from human umbilical cord mesenchymal stem cells using Trizol reagent (TransGen Biotech, Beijing, China). With iScript RT-PCR Kit (Bio-Rad, California, USA), 1 μg of purified RNA was used for reverse transcription. QPCR analysis was performed using SYBR Green PCR Master Mix (Bio-Rad, California, USA). The expressions of 18S, PGC-1α, Nrf2 and Sirt3 were detected by real-time quantitative PCR. The expression of the target gene was calculated using the 2-^△△^Ct method and normalized to 18S.

### Immunocytofluorescense staining and confocal imaging

Human umbilical cord mesenchymal stem cells were seeded on coverslips. After treatment, cells were fixed in −20 °C methanol, permeabilized in 0.1% Triton X-100 for 15 min, and blocked with 1% PBS-inactivated fetal bovine serum (Hyclone, Logan, USA) at room temperature for 30 min. Primary antibody (1∶400; 3033T, Cell Signal Technology) with rabbit anti-phosphorylated NF-κBp65 antibody was incubated overnight at 4 °C. Cells were washed three times with PBS. Incubate with FITC-labeled donkey anti-rabbit secondary antibody (1∶1000, ab6798, Abcam) in the dark at room temperature for 2 h. Nuclei were stained with 0.5 μg/mL of 4',6-di-2-yl-2-phenylindole (DAPI, Beyotime, C1002) for 15 min. Image the slides using a confocal microscope (A1, Nikon, Japan).

### Date and statistical analysis

Statistical analysis was performed with SPSS 22.0. Normal distribution data were expressed as mean ± standard deviation (SD). Skewed distribution statistics was expressed as median (25th–75th percentile). Differences between two groups were analyzed by Student's *t* test while one-way analysis of variance for three or more groups. For all statistical analyses, statistical significance was considered as *p* < 0.05.

## Abbreviations

**Table 1:** 

BP	Biological processes
CC	Cellular components
GO	Gene ontology
HUC-MSCs	Human umbilical cord mesenchymal stem cells
KEGG	Kyoto encyclopedia of genes and genomes
MF	Molecular functions
MSCs	Mesenchymal stem cells
MTs	Metallothioneins
Nrf2	Nuclear factor erythroid-2-related factor 2
ROS	Reactive oxygen species
Sirt3	Sirtuin 3
PGC-1α	Peroxisome proliferator-activated receptor gamma co-activator 1α

**Table 2:** 

SLC	Solute carrier transporters
TPEN	N,N,N’,N’-tetrakis-(2-pyridylmethyl)-ethylenediamine
MTT	3-(4,5-dimethylthiazol-2-yl)-2,5-diphenyltetrazolium bromide
Zn	Zinc

## Conflict of interest

Xiaodan Lu, Yifan Lin, Xiuying Lin, Qiang Zhang, Zihang Wang, Xuguang Mi, Ruobing Wang, Xiaofang Zhang, Xu Luan, Yan Liu, Bing Li, Yan Tan and Yanqiu Fang declare that they have no conflict of interest.
